# Comparing the Performance of Popular Large Language Models on the National Board of Medical Examiners Sample Questions

**DOI:** 10.7759/cureus.55991

**Published:** 2024-03-11

**Authors:** Ali Abbas, Mahad S Rehman, Syed S Rehman

**Affiliations:** 1 Medical School, University of Texas Southwestern Medical School, Dallas, USA; 2 Nephrology, Baptist Hospitals of Southeast Texas, Beaumont, USA

**Keywords:** chatgpt, google's bard, gpt-4, claude, artificial intelligence and education, artificial intelligence in medicine, united states medical licensing examination (usmle), nbme subject exam, large language model, artificial intelligence (ai)

## Abstract

Introduction: Large language models (LLMs) have transformed various domains in medicine, aiding in complex tasks and clinical decision-making, with OpenAI's GPT-4, GPT-3.5, Google’s Bard, and Anthropic’s Claude among the most widely used. While GPT-4 has demonstrated superior performance in some studies, comprehensive comparisons among these models remain limited. Recognizing the significance of the National Board of Medical Examiners (NBME) exams in assessing the clinical knowledge of medical students, this study aims to compare the accuracy of popular LLMs on NBME clinical subject exam sample questions.

Methods: The questions used in this study were multiple-choice questions obtained from the official NBME website and are publicly available. Questions from the NBME subject exams in medicine, pediatrics, obstetrics and gynecology, clinical neurology, ambulatory care, family medicine, psychiatry, and surgery were used to query each LLM. The responses from GPT-4, GPT-3.5, Claude, and Bard were collected in October 2023. The response by each LLM was compared to the answer provided by the NBME and checked for accuracy. Statistical analysis was performed using one-way analysis of variance (ANOVA).

Results: A total of 163 questions were queried by each LLM. GPT-4 scored 163/163 (100%), GPT-3.5 scored 134/163 (82.2%), Bard scored 123/163 (75.5%), and Claude scored 138/163 (84.7%). The total performance of GPT-4 was statistically superior to that of GPT-3.5, Claude, and Bard by 17.8%, 15.3%, and 24.5%, respectively. The total performance of GPT-3.5, Claude, and Bard was not significantly different. GPT-4 significantly outperformed Bard in specific subjects, including medicine, pediatrics, family medicine, and ambulatory care, and GPT-3.5 in ambulatory care and family medicine. Across all LLMs, the surgery exam had the highest average score (18.25/20), while the family medicine exam had the lowest average score (3.75/5).

Conclusion: GPT-4's superior performance on NBME clinical subject exam sample questions underscores its potential in medical education and practice. While LLMs exhibit promise, discernment in their application is crucial, considering occasional inaccuracies. As technological advancements continue, regular reassessments and refinements are imperative to maintain their reliability and relevance in medicine.

## Introduction

Advances in artificial intelligence (AI), particularly the development of large language models (LLMs), have revolutionized numerous fields, including medicine. AI has found its way into diverse medical specializations, including oncology, radiology, and pathology, showcasing its evolving clinical uses [1]. These advanced models assist healthcare professionals in complex tasks, from cancer detection and categorization to adjusting insulin regimens in diabetic patients [2-4]. As the development of new models persists, AI will continue to revolutionize our comprehension and approach to medicine. As of June 2023, over 20 LLMs are available for public use, with OpenAI's GPT-4, GPT-3.5, Google’s Bard (based on PaLM 2), and Anthropic’s Claude among the most widely used [5].

Although previous studies have shown the ability of individual LLMs to pass certain medical licensing exams [6], there is limited research comparing the performance of different LLMs. Most recently, GPT-4 has shown to outperform other LLMs in answering questions relating to various medical specialties, such as neurosurgery, orthopedics, and general surgery [7-9]. Furthermore, GPT-4 outperformed its predecessor, ChatGPT, on the United States Medical Licensing Exam (USMLE) soft skills exam [10], displaying its capacity for empathy in addition to technical knowledge. Considering this potential, there is growing interest in assessing the capabilities of LLMs in medical education and practice. When students or educators use LLMs, it is important to know which LLM would provide the most accurate information in a broad variety of subjects.

The National Board of Medical Examiners (NBME) clinical subject exams serve as an important measure of a medical student's knowledge and clinical capabilities [11-12]. The NBME subject exams are taken by medical students, typically at the conclusion of a clinical rotation in the respective field. For example, a neurology subject exam would be taken by a medical student during their neurology clerkship rotation. This study aims to compare the performance of popular LLMs on sample questions from the NBME clinical subject exams. Given the capabilities of LLMs, the outcomes of this study could highlight the potential advantages of certain LLMs over others.

## Materials and methods

This study was a comparative analysis, aiming to evaluate the performance of various LLMs in answering medical multiple-choice questions. The questions used in this study were multiple-choice questions obtained from the official NBME website and are free and publicly available [13]. Questions from the NBME clinical science subject exams in medicine, pediatrics, obstetrics and gynecology, clinical neurology, ambulatory care, family medicine, psychiatry, and surgery were used along with questions from the NBME sample comprehensive clinical science exam. Each exam has a set of sample questions consisting of either 19 or 20 questions, with the exception of family medicine, which has five questions. All questions from each exam were used and no questions were omitted.

The LLMs queried include GPT-4, GPT-3.5, Claude, and Bard. None of the LLMs had Internet access enabled. All data were collected in October 2023. For consistency, each NBME question, including its lettered answer choices, was individually inputted into the designated text field of each LLM studied with no additional text in the prompt. Using a systematic approach, each question was inputted individually in the order in which they were presented on the NBME sample exam, and all parts of each question were provided as they appeared on the exam. Each LLM was prompted with each individual question one time. All questions were multiple-choice-style questions with one correct answer. The answer choice provided in the response from each LLM was recorded and compared to the correct answer provided by NBME. The total number of questions answered correctly by each LLM was recorded.

The one-way analysis of variance (ANOVA) was used to determine statistically significant differences between LLM performance for each subject and overall performance. In situations where the ANOVA indicated significant differences, a post-hoc analysis was conducted using the Bonferroni correction to adjust for multiple comparisons, with the significance level set at alpha <0.0125. All statistical evaluations were performed using the R statistical software, specifically version 3.5.1 (R Foundation for Statistical Computing, Vienna, Austria).

## Results

**Figure 1:**
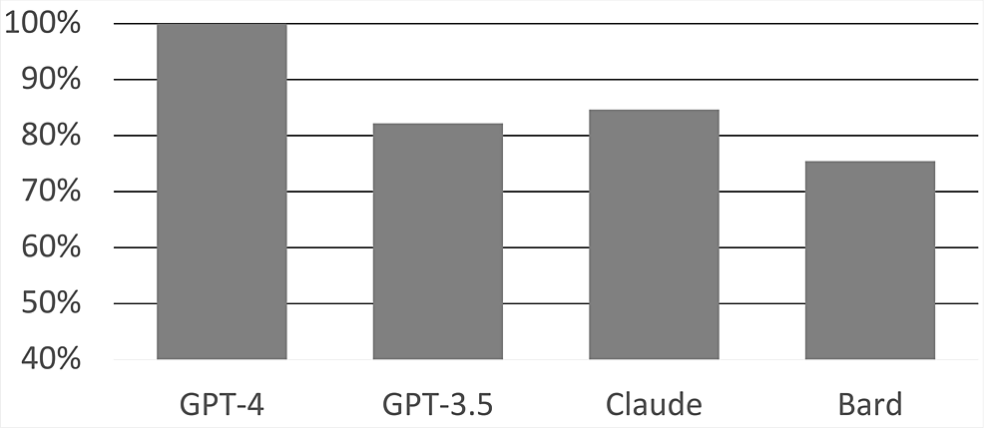
Overall performance of the LLMs on all NBME sample questions LLM: large language model, NBME: National Board of Medical Examiners

A total of 163 questions were queried by each LLM. GPT-4 scored 163/163 (100%), GPT-3.5 scored 134/163 (82.21%), Claude scored 138/163 (84.66%), and Bard scored 123/163 (75.46%) (Figure 1). In terms of total performance, GPT-4 significantly outperformed GPT-3.5 by 17.8% (p < 0.001), Claude by 15.3% (p < 0.001), and Bard by 24.5% (p < 0.001). No significant difference was observed between the total performance of GPT-3.5, Claude, and Bard. 

**Figure 2:**
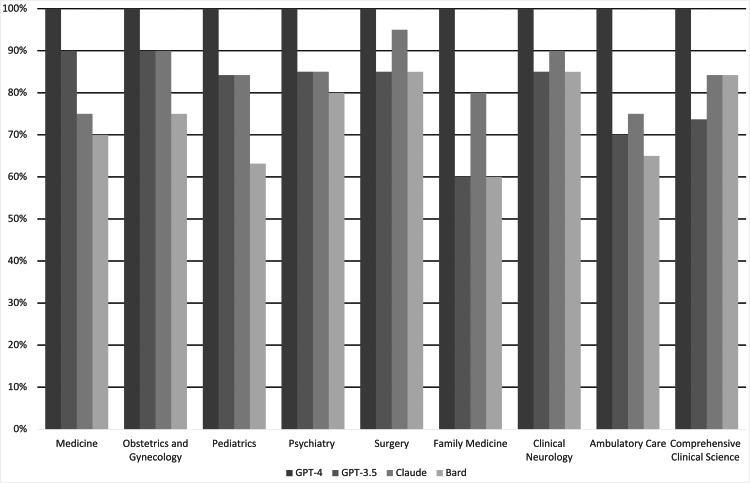
Performance of the LLMs on each subject exam LLM: large language model

GPT-4 scored 100% on all subject exams. GPT-3.5 performed best on the medicine and obstetrics/gynecology exams (18/20) and lowest on the family medicine exam (3/5). Claude performed best on the surgery exam (19/20) and lowest on the ambulatory care exam (15/20). Bard performed best on the surgery and neurology exams (17/20) and lowest on the family medicine exam (3/5). Comprehensive results are depicted in Table 1 and Figure 2. 

**Table 1:** Number of questions answered correctly by the LLMs on each subject exam LLM: large language model

	Internal Medicine	Obstetrics and Gynecology	Pediatrics	Psychiatry	Surgery	Family Medicine	Clinical Neurology	Ambulatory Care	Comprehensive Clinical Science
GPT-4	20	20	19	20	20	5	20	20	19
GPT-3.5	18	18	16	17	17	3	17	14	14
Bard	14	15	12	16	17	3	17	13	16
Claude	15	18	16	17	19	4	18	15	16
Total Questions	20	20	19	20	20	5	20	20	19

GPT-4 significantly outperformed Bard on medicine, pediatrics, family medicine, and ambulatory care (p < 0.01). GPT-4 also significantly outperformed GPT-3.5 on ambulatory care and family medicine (p < 0.01). There was no statistically significant difference in the performance of GPT 3.5, Claude, and Bard among the different subject exams. The highest average score of all LLMs on a single subject exam was on the surgery exam (18.25/20), and the lowest average score of all LLMs on a single subject exam was on the family medicine exam (3.75/5). The average score received across all LLMs for each subject exam is depicted in Table 2.

**Table 2:** Average score across all the LLMs on each NBME subject exam LLM: large language model, NBME: National Board of Medical Examiners

	Medicine	Obstetrics and Gynecology	Pediatrics	Psychiatry	Surgery	Family Medicine	Clinical Neurology	Ambulatory Care	Comprehensive Clinical Science
Average Score	16.75/20	17.75/20	15.75/19	17.5/20	18.25/20	3.75/5	18/20	15.5/20	16.25/19
Average Percent Correct	83.75%	88.75%	82.89%	87.50%	91.25%	75%	90%	77.50%	85.53%

## Discussion

This study presents a comprehensive analysis comparing the performance of prominent LLMs on sample questions from the NBME clinical subject exams. GPT-4 performed the best, achieving a perfect score of 100% across all questions. One potential reason for GPT-4's standout performance may be its vast training data, exceeding 45 terabytes by September 2021 [14]. The model, despite not being specifically fine-tuned for medical data, demonstrated an ability to respond to intricate medical queries [15]. OpenAI has vast amounts of data on its website that showcases GPT-4 outperforming GPT-3.5 across different disciplines, including law, language, math, and social and political studies [14]. The findings of this study expand on the data provided by OpenAI to include GPT-4's superiority over GPT-3.5 in answering questions stemming from clinical vignettes related to various subjects within medicine. 

GPT-3.5, Bard, and Claude, despite their respectable performances, were outpaced by GPT-4. This could hint at the importance of the sheer volume and diversity of training data seen in GPT-4. However, it is also essential to consider other factors like model architecture, fine-tuning strategies, and the nature of the questions in the exams [16]. The results underline the potential of LLMs in medical education and practice. Their capabilities in answering intricate medical questions could pave the way for innovative applications in clinical decision support, research, and education. Although this study provides support for the use of GPT-4 over other LLMs in the everyday practice of students and educators, it is the only LLM in this study that is not free to use, which could be a barrier to the use by a large proportion of students [17].

The remarkable performance of GPT-4 in responding to medical queries underscores the growing significance of LLMs in healthcare, resonating with emerging literature on AI applications in diagnostics and clinical practice. Recent studies have detailed the landscape of FDA-approved AI devices and algorithms, suggesting an accelerating integration of AI in medical diagnostics and patient care and reflecting a broader trend of digital transformation in healthcare [18]​​. Similarly, deep learning models have improved diagnostic accuracies in breast cancer screening, highlighting the potential of this technology to enhance the precision of medical diagnoses alongside human experts [19,20]​​. Moreover, the transformative impact of AI extends beyond diagnostics. Recent work has shown that AI can interpret complex medical data, such as echocardiograms and cardiac function assessments, with high accuracy, suggesting its utility in cardiology​​ [21,22]. Furthermore, studies have shown that AI can support early lung cancer detection, potentially improving patient outcomes [23]. These advancements signify a shift toward more AI-integrated medical practices, promising improved efficiencies and patient outcomes across various medical specialties.

Future research on the application of LLMs in medicine should aim to deepen our understanding of their potential and limitations. This includes exploring a broader array of AI models, particularly those specialized in distinct medical fields or capable of interpreting complex medical imagery, to uncover models that excel in specific areas. Expanding the diversity of medical subjects on which LLMs are trained is also vital, as studies have shown that current algorithmic-based systems may reinforce gender biases and affect marginalized communities in healthcare-related applications [24]. Longitudinal studies would also be valuable in tracking the progress and adaptation of LLMs over time, shedding light on their learning curves, improvements in accuracy, and ability to integrate new medical knowledge. Such research efforts will not only enhance the utility of LLMs in clinical and educational settings but also contribute to safer, more effective healthcare delivery.

While LLMs display promise, it is vital to approach their use with caution. LLMs can occasionally provide answers with unwarranted confidence, even if incorrect [25]. Therefore, while they can be invaluable tools, clinicians and students must critically evaluate their responses. Any implementation of LLMs in a clinical setting must prioritize patient safety and the validation of the information provided [26,27].

In addition to the demonstrated capabilities of LLMs, it is pivotal to acknowledge this study's limitations. Notably, the absence of sample questions from the NBME featuring an image or other media components, prevalent in real exams, may pose a limitation to the external validity of our findings. Our sample size was also limited due to a limited number of publicly available NBME sample questions, which can affect the generalizability of our results. In addition, our study did not explore the efficiency of LLMs when exposed to real-time clinical scenarios, which often encompass multifaceted clinical reasoning and not just recall-based knowledge [28]. Moreover, while GPT-4's extensive training data undoubtedly contributes to its superiority, the inability to assess it against niche, specialized medical models could limit our understanding of its true potential within the medical field. Lastly, as technological advancements march forward, it is worth noting that models like GPT-4 might become outdated, emphasizing the continuous need for reassessment and adaptation in this rapidly advancing field.

## Conclusions

GPT-4 demonstrated superior performance, achieving a remarkable 100% accuracy rate in responding to sample questions from NBME clinical subject exams, significantly surpassing other LLMs, such as GPT-3.5, Claude, and Bard. Overall, the LLMs performed best on sample questions from the surgery clinical subject exam and worst on sample questions from the family medicine clinical subject exam.

Despite these promising results, the study emphasizes the necessity for cautious integration of LLMs into medical practice and education. Given the rapid evolution of AI technologies and their potential implications for patient safety and information validity, critical appraisal by healthcare professionals and educators is imperative. The study acknowledges limitations, including the exclusion of image-based questions and reliance on a limited pool of publicly available questions, potentially affecting the findings' generalizability.

This research contributes significantly to the literature on AI in healthcare, outlining both the capabilities and challenges associated with the use of LLMs in medical education and practice. It underscores the transformative potential of AI in the medical domain while advocating for a balanced approach to ensure the ethical and safe application of these technologies, underscoring the need for continuous evaluation and validation to effectively harness the full potential of LLMs in healthcare.
